# Some False Impressions Corrected

**Published:** 1882-06

**Authors:** 


Some False Impressions Corrected. — Most readers of a medical journal seem to think that the existence of the editor is a very blissful one ; that he generally sits in a comfortable arm-chair in a cosy little apartment known as his *sanctum ;* that most of his time is occupied in contemplating with satisfaction a large paid-up subscription list, and that in the intervals when not so engaged he dashes off with the greatest ease editorials upon the "Responsibility of Guiteau" or the "Development of the Color Sense."

It is unkind to dissipate such a rose-colored vision, but in the interests of truth, to the diffusion of which The Independent Practitioner is devoted, we are compelled to correct such false impressions. In the majority of cases the medical editor is a poor devil who has been victimized by misplaced confidence. Misled by a large subscription list, he comes to believe that subscriptions represent cash, and it is not until he finds at the end of the year that the greater figures are at the bottom of the debit column, that he begins to doubt. We are sure our readers want their first impression—the *couleur de rose* view—of the editor's life to be the true one. Hence we would suggest a cheerful compliance with our hint of their obligation enclosed in the April number. A prompt remittance will relieve us of the embarrassment of asking for what is still due from many of our subscribers. We would be under especial obligations to any one who may inform us of any mistake in his bill or address. All errors will be promptly corrected if our attention is directed to them.

